# Colorectal image analysis for polyp diagnosis

**DOI:** 10.3389/fncom.2024.1356447

**Published:** 2024-02-09

**Authors:** Peng-Cheng Zhu, Jing-Jing Wan, Wei Shao, Xian-Chun Meng, Bo-Lun Chen

**Affiliations:** ^1^Faculty of Computer and Software Engineering, Huaiyin Institute of Technology, Huaian, China; ^2^Department of Gastroenterology, The Second People's Hospital of Huai'an, The Affiliated Huai'an Hospital of Xuzhou Medical University, Huaian, Jiangsu, China; ^3^Nanjing University of Aeronautics and Astronautics Shenzhen Research Institute, Shenzhen, China; ^4^Department of Physics, University of Fribourg, Fribourg, Switzerland

**Keywords:** medical data mining, medical intelligence, colorectal cancer, polyp diagnosis, attention mechanism, medical image detection

## Abstract

Colorectal polyp is an important early manifestation of colorectal cancer, which is significant for the prevention of colorectal cancer. Despite timely detection and manual intervention of colorectal polyps can reduce their chances of becoming cancerous, most existing methods ignore the uncertainties and location problems of polyps, causing a degradation in detection performance. To address these problems, in this paper, we propose a novel colorectal image analysis method for polyp diagnosis via PAM-Net. Specifically, a parallel attention module is designed to enhance the analysis of colorectal polyp images for improving the certainties of polyps. In addition, our method introduces the GWD loss to enhance the accuracy of polyp diagnosis from the perspective of polyp location. Extensive experimental results demonstrate the effectiveness of the proposed method compared with the SOTA baselines. This study enhances the performance of polyp detection accuracy and contributes to polyp detection in clinical medicine.

## 1 Introduction

Colorectal cancer (CRC), as a serious malignancy, has been a major public health challenge worldwide. Its high morbidity and mortality rates have made it an important focus of healthcare systems in many countries. It is one of the most common malignant tumors in clinical practice, and the number of people suffering from it is on the rise every year (Soons et al., 2022). At present, with the change in people's lifestyles and irregular diet structure, the incidence of colorectal cancer is getting closer to the younger age group, which brings great torment to the physical and mental health of many people. According to the data from the World Health Organization (WHO) and the International Agency for Research on Cancer (IARC), nearly 2 million people are diagnosed with colorectal cancer every year, and about 800,000 people have been taken away from their lives, and the number is rising every year (Sninsky et al., 2022). Therefore, how to avoid colorectal cancer and timely diagnosis is crucial.

Fortunately, CRC is often potentially preventable and early diagnosed. Prompt diagnosis and effective treatment in the early stages of colorectal cancer can reduce its incidence. The evolution of colorectal cancer is a multi-step process. In the early stages, most colorectal cancers usually appear as polyps, especially adenomatous polyps. Therefore, early detection and removal of colorectal polyps can effectively prevent CRC. According to data, the removal of colorectal polyps at an early stage can reduce the mortality associated with CRC by up to 70% (Barua et al., 2021).

In clinical medicine, colonoscopy is considered to be the most direct and effective means of detecting polyps and is regarded as the gold standard for reducing the incidence of colorectal cancer. The effectiveness of colonoscopy in preventing colorectal cancer depends mainly on the detection rate of polyps. Evidence suggests that for every 1.0% increase in the detection rate of colorectal polyps, the associated incidence of CRC can be reduced by 3.0%–6.0% (Sinonquel et al., 2021). However, the quality of colonoscopy is susceptible to several factors, including the skill of the endoscopist, the light conditions of the endoscope, and the quality of bowel cleansing that may lead to a decrease in the detection rate of polyps. According to incomplete statistics, the polyp leakage rate of colonoscopy is as high as 27%, which makes polyp detection difficult to achieve the expected results (Hassan et al., 2021). Therefore, some scholars seek some other means to increase the polyp detection rate of colonoscopy, thus reducing the incidence of CRC.

In recent years, the field of artificial intelligence (AI) has been booming, and computer-aided diagnosis (CAD) systems based on deep learning are advancing and bringing many potential benefits to the medical field. The use of AI methods to assist colorectal cancer polyp examination can not only overcome the limitations of traditional colonoscopy, improve the accuracy of polyp detection, and reduce the rate of leakage, but also reduce the clinician's workload, improve work efficiency, and promote the development of the medical field toward intelligence.

Despite the current remarkable results of deep learning in colonoscopy, there are still some issues to be explored. For example, the amount of data is insufficient: deep learning algorithms generally require a large amount of labeled data for training to improve the accuracy of the model (Ng et al., 2020). However, there are data privacy, ethical issues, and legal issues associated with the acquisition of endoscopic data, making it a challenge to acquire large-scale endoscopic labeling data. Lack of sufficient labeled data may limit the performance of model algorithms. Interpretability of the model: the decision-making process of deep learning models is difficult to interpret. This is an important issue for the medical field. The interpretability of deep learning models also limits their wide application in clinical practice. Detection performance: the detection performance exhibited by the model in different environments still needs to be improved (Tajbakhsh et al., 2015). Therefore, how to design a detection method that can accurately extract polyp features and exhibit high accuracy and low complexity under limited endoscopic labeling data resources is now a hot research topic.

This paper discusses how to reduce the leakage rate of colorectal cancer polyp detection from the perspective of deep learning. This can not only improve the polyp detection rate in endoscopy and colorectal cancer diagnosis and treatment, but also improve the efficiency of endoscopists. The main contributions of this paper include:

1. A new attention mechanism module was designed to focus on the semantic information of polyps in the polyp feature extraction stage;
2. The GWD loss is introduced to improve the regression accuracy of the target frame, thus reducing the polyp detection leakage rate.

## 2 Related works

Currently, object detection algorithms are mainly categorized into traditional target detection algorithms and deep learning-based detection algorithms. The traditional detection model mainly contains three components: region selection, feature extraction, and classifier classification. It mainly relies on manually designed feature extractors and machine learning algorithms. However, the traditional algorithms face the difficulty of manual feature design, sensitivity to image changes, and difficulty in coping with complex tasks.

In the past decade or so, how to achieve automated polyp detection has been an active topic of research in the medical field, and a lot of research has been done to develop related technologies and algorithms. In recent years, the application of deep learning techniques in medical imaging has become increasingly popular. Among them, research on polyp detection using deep learning has also received much attention. It can be categorized into two-stage and one-stage detection based on the detection steps. Two-stage detection is known for its high accuracy, but the inference speed is slow and the real-time performance is poor. Single-stage adopts the idea of regression and uses CNN to directly perform location localization for the classification of polyps, which is an end-to-end algorithm with fast detection speed and slightly lower accuracy.

### 2.1 Two-stage polyp detection model

Taş and Yılmaz (2021) improved the model based on Faster R-CNN by first using ResNet-101 instead of VGG16 and then using the super-resolution based convolutional neural network (SRCNN) method to improve the resolution of colonoscopy images and finally improve the accuracy of the model. Cheng et al. (2021) improved Mask R-CNN based on Mask R-CNN and firstly proposed a learnable directed derivative network (LOD-Net) to calculate eight directed derivatives for each pixel of polyps, and then used these derivative values to form a candidate frame for polyps, which ultimately improved the accuracy of polyp detection regression. He et al. (2023) proposed a two-stage polyp detection algorithm, they designed a two-stage detection algorithm called UY-Net by combining U-Net and YOLOv4. Experiments proved that the accuracy of UY-Net is higher than that of mainstream detection algorithms such as YOLOv4 and YOLOv3-SPP. Yang et al. (2020) proposed an improved Mask R-CNN for instance segmentation of endoscopic polyp lesion areas. The method is divided into two parts, first filtering the original image to remove the noise in the image, and then feeding the processed image into the improved Mask R-CNN. Experimental results demonstrate the effectiveness of the method. Jia et al. (2020) proposed a two-stage feature pyramid network based on deep learning for the detection of endoscopic polyp lesion regions, first extracting polyp features in the first stage with a Faster R-CNN network and then using feature sharing to feed the feature learned in the first stage into the polyp detection task in the second stage, making the polyp detection more accurate. Dermane and Torch (2023) propose to use ab initio neural network training to allow the network to learn features from given polyp data. Then, migration learning was performed using the pre-trained VGG19. Finally, the ab initio trained network was combined with VGG19, ResNet50, and Inceptionv3 models. The results show that this approach improves the overall accuracy (Dermane and Torch, 2023). Ribeiro et al. (2016) used a convolutional neural network (CNN) to detect colorectal polyps, which directly uses the input polyp image pixels to extract features and classify the images in the same architecture. The method was shown to be effective in handling distortions in the presence of different lighting conditions, presence of partial occlusions, etc. (Ribeiro et al., 2016).

Currently, the two-stage polyp detection algorithm model based on deep learning has achieved the best performance in terms of accuracy. However, its high temporal complexity makes it difficult to meet the real-time nature of polyp detection in the field of clinical medicine, and therefore, it is difficult to realize large-scale applications.

### 2.2 Single-stage polyp detection model

Laddha et al. (2019) implemented the detection of polyps using two models of different sizes, YOLOv3 and YOLOv3-tiny, based on varying polyp sizes, ultimately obtaining 91% and 82% accuracy. Cao et al. (2021) designed a feature extraction and fusion module based on yolov4 and combined it with the yolov4 network to solve the problem of inconspicuous contrast between polyps and background during detection. Finally, 91.6% was achieved in terms of accuracy (Cao et al., 2021). Yu et al. (2022) designed the ITH module based on Scaled-YOLOv4, which shares weights with the YOLO detection head for fast feature extraction. In addition, a learning method based on similarity metrics is designed to improve the performance of model evaluation. The introduction of the ITH module enables the model to improve the recognition speed by 30% compared to the original model (Yu et al., 2022). Dash et al. (2023) proposed an expert system designed to address the problems of time-consuming polyp detection and high misdiagnosis rates. The system uses an unsupervised deep belief network (DBN) to extract effective polyp features. The network was experimentally demonstrated to help improve the accuracy of polyp detection (Dash et al., 2023). Shin et al. (2018) proposed a region-based convolutional neural network for the presence of false polyps during detection. Based on this, two further post-learning methods, offline learning, and automatic false positive learning, are proposed. Experimental data show that this method exhibits better performance in detecting colonoscopy video streams (Shin et al., 2018). Pacal et al. (2022) proposed a method to integrate Cross Stage Partial Network (CSPNet) in YOLOv3 and YOLOv4 to achieve real-time polyp detection with guaranteed high detection rates. The method also uses data augmentation and migration learning strategies to improve polyp detection performance (Pacal et al., 2022). Nogueira-Rodŕıguez et al. (2022) designed a real-time polyp detection model that uses a pre-trained YOLOv3 architecture and follows up with an object-tracking algorithm. The method can be effectively integrated into CAD systems (Nogueira-Rodŕıguez et al., 2022). Karaman et al. (2023) proposed to integrate the Artificial Bee Colony algorithm (ABC) into the Scaled-YOLOv4 model and evaluated it on the SUN and PICCOLO polyp datasets. The method was experimentally found to be useful in improving the model's mAP and F1-score metrics for polyp detection (Karaman et al., 2023). Zhang et al. (2019) designed a new model known as SSD-GPNet. The model takes into account the polyp features discarded in the pooling layer and connects them as additional feature maps to help the model for detection. At the same time, the bottom feature maps in the feature pyramid are connected to the top feature maps to establish explicit relationships between the layers. Experiments show that the SSD-GPNet model exhibits satisfactory performance in detecting small polyps (Zhang et al., 2019). Lalinia and Sahafi (2023) proposed an artificial intelligence-based polyp detection system using the YOLOv8 network model and constructed a diverse dataset for training. Ahmad et al. propose an automated polyp detection method. The method enhances the performance of the YOLOv7 object detection algorithm by the integration of a Squeeze and Excitation attention block. This integration greatly improves polyp detection with favorable results (Ahmad et al., 2023). Khryashev et al. (2023) proposed to solve the problem of a low number of images in the dataset by data augmentation and used the YOLOv8 model for colorectal polyp detection.

Although single-stage target detection achieves an advantage in computation time, it is prone to false positives and false negatives in the face of the complexity of the intestinal environment in colonoscopy. In addition, the performance of single-stage detection algorithms is still unsatisfactory when facing polyps with low contrast to the background and smaller polyps. Therefore, designing a high-accuracy polyp detection algorithm with real-time performance has been a hot research topic in the medical field.

## 3 Methods

### 3.1 The model for polyp detection

**Figure 1:**
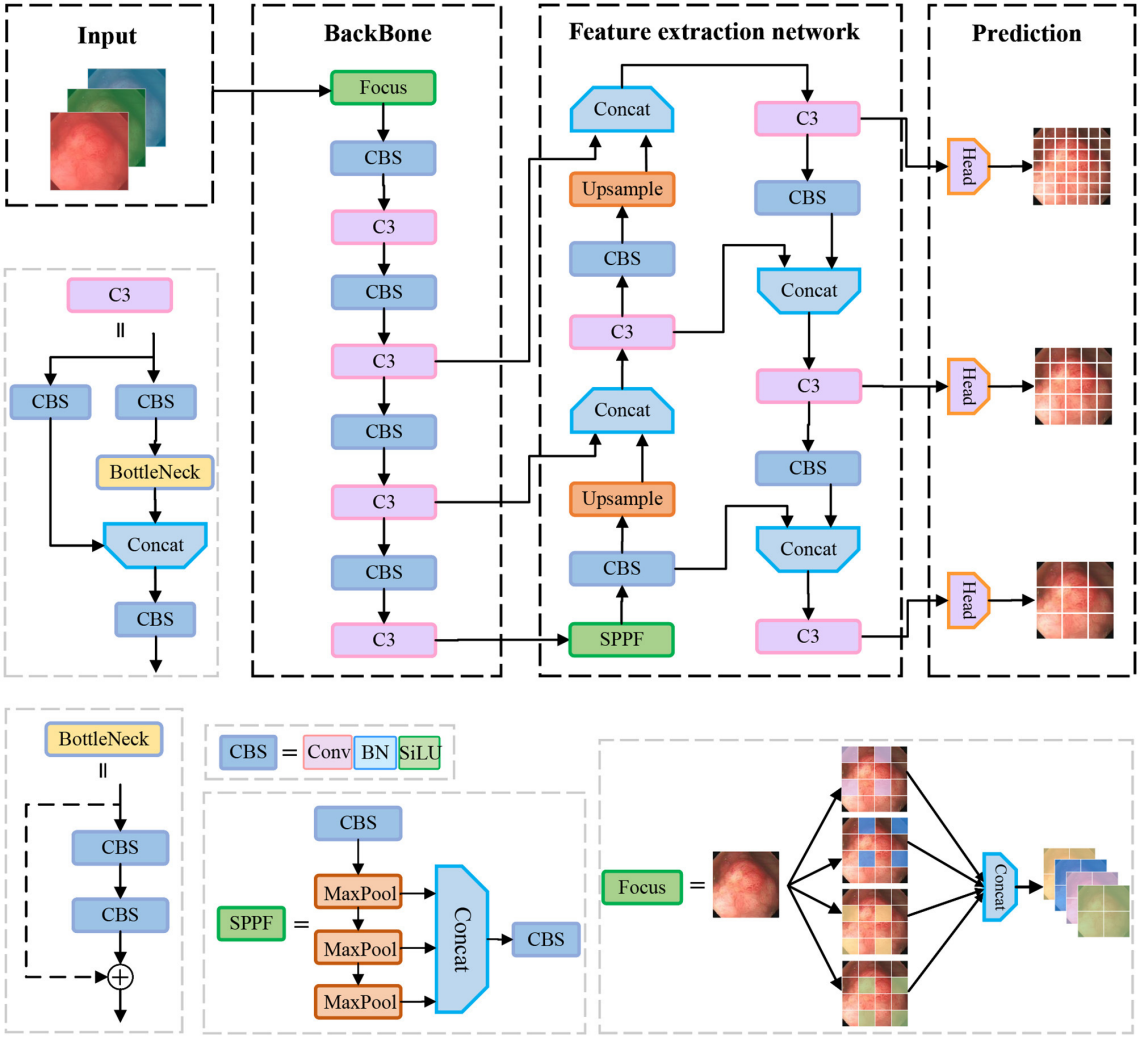
Illustration of the architecture of the original YOLOv5 model for polyp detection.

In the field of object detection, YOLOv5 is one of the most widely recognized one-stage object detection algorithms, which has a high detection speed along with detection accuracy. In this paper, we propose to use YOLOv5 as a benchmark model for polyp detection. Its network structure is shown in Figure 1, which is mainly composed of the following four parts: Input, Backbone, Neck, and Head.

**Figure 2:**
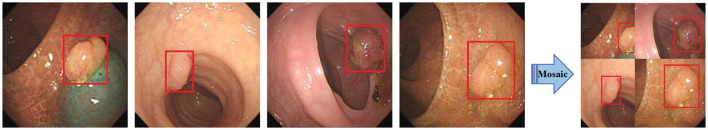
Mosaic data enhancement diagram. The red squares in the figure are the bounding boxes of the polyps.

Input: The input side preprocesses the input data. Data enhancement and adaptive anchor framing are mainly accomplished in this stage. YOLOv5 integrates a large number of conventional data enhancement methods, such as graphic transformation, hue adjustment, etc. In addition, data enhancement methods such as Mosaic and Mixup are also included, which greatly enriches the amount of data. The Mosaic data enhancement effect is shown in Figure 2. Since the optimal anchor frame hyperparameters differ from one dataset to another, YOLOv5 is based on the K-means algorithm to adaptively obtain the anchor frame hyperparameters that best fit the dataset.

Backbone: The CSPDarknet53 is used as the backbone network of yolov5, which consists of 53 convolutional layers for extracting features from images. The network gradually reduces the resolution and captures different levels of feature information for detecting targets of different sizes. In addition, it uses residual links to avoid the problem of gradient vanishing while training the network. In the backbone network, the C3 structure and the SPPF structure are used. The C3 module consists of three convolution units and a Bottleneck module. Firstly, the input feature maps are convolved by two separate 1 × 1 convolutions. Then, the output of one convolution unit is input into the Bottleneck module and the output features of the other convolution unit are spliced in the channel dimension. Finally, the features are output through a convolution unit. The motivation of the C3 is to increase the depth and receptive field of the network and improve the ability of the network to extract polyp features. The SPPF structure stacks three identical max-pooling layers of convolution kernel size 5 × 5 in series, which further increases the receptive field by successive max-pooling.

Neck: The neck part of the yolov5 network is mainly responsible for fusing the feature information of different layers in the backbone network to enhance the network model's ability to detect objects of different sizes. It uses FPN (Feature Pyramid Network) and PAN (Path Aggregation Network) structures to construct top-down and bottom-up information pathways, passing strong semantic features from the upper layer to the lower layer, and then passing detailed features from the lower layer to the upper layer so that the feature layers of different resolutions have both semantic and detailed information.

Head: The head part is responsible for outputting the results of detecting the object, including the position information and category information of the object.

In the process of polyp detection, despite the remarkable detection accuracy and speed of yolov5, the weak contrast between the target to be detected and the background in the process of polyp detection makes the model still have some challenges in polyp feature extraction. Therefore, to better reduce the leakage rate of polyps to improve the detection accuracy and the accuracy of bounding box regression, this paper proposes to improve the detection accuracy of polyps by improving the network structure of YOLOv5 and the accuracy of bounding box localization by modifying the loss function.

### 3.2 Attention mechanism for detection accuracy

The attention mechanism is to focus on key areas and suppress feature information in non-target areas by assigning different weight values to different parts of the feature map, to achieve the purpose of extracting better features. Woo et al. (2018) combined spatial attention and channel attention to design a convolutional block attention module known as CBAM. The attention first extracts global features channel by channel generates channel attention features, uses the features as inputs to the spatial attention structure, and finally generates a hybrid domain feature map.

**Figure 3:**
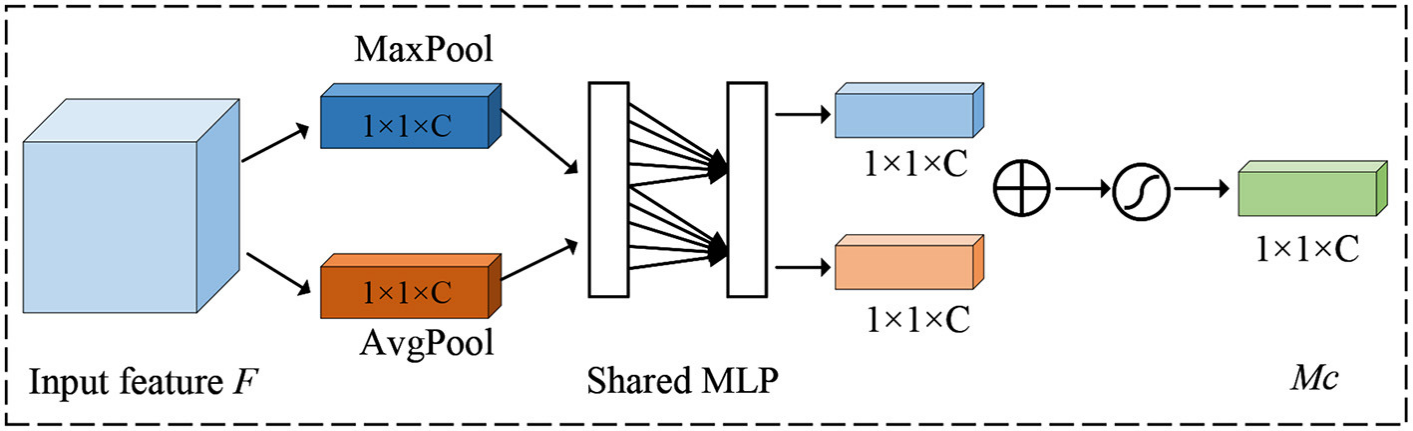
Illustration of the architecture of the channel attention module.

For channel attention, its purpose is to enhance the model's perception of features on different channels. It mainly adjusts the feature map by considering the importance of each channel. The detailed details of the channel attention mechanism are shown in Figure 3.

Firstly, a global average pooling operation is done for each channel to obtain the mean value over the channel dimension, thus obtaining a global context vector that represents the information of the entire feature map channel. Next, the global context vector is transformed into a vector of channel attention weights, where each element corresponds to a channel, under the action of full connectivity. Finally, the channel attention weights are multiplied with the original feature map to enhance the responses of important channels and suppress the responses of non-critical channels.

**Figure 4:**
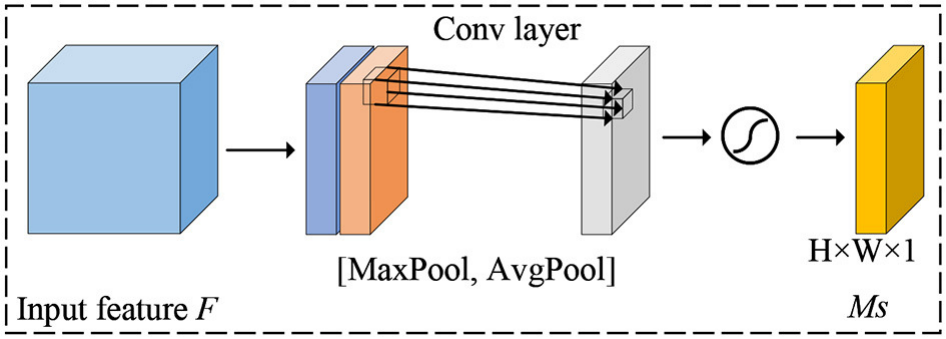
Illustration of the architecture of the spatial attention module.

For spatial attention, its purpose is to enhance the model's perception of feature responses at different locations. It mainly adjusts the feature map by considering the importance of each pixel point. The detailed details of the spatial attention mechanism are shown in Figure 4.

First, for each channel, a global maximum pooling operation is performed to obtain the maximum value in the channel dimension, which results in a global context vector representing the spatial information of the entire feature map. Next, the global context vector is transformed into a spatial attention weight map using full connectivity, where each pixel point corresponds to a location. Finally, the spatial attention weights are multiplied with the original feature map to enhance the feature responses at important locations and suppress the feature responses at non-critical locations.

**Figure 5:**
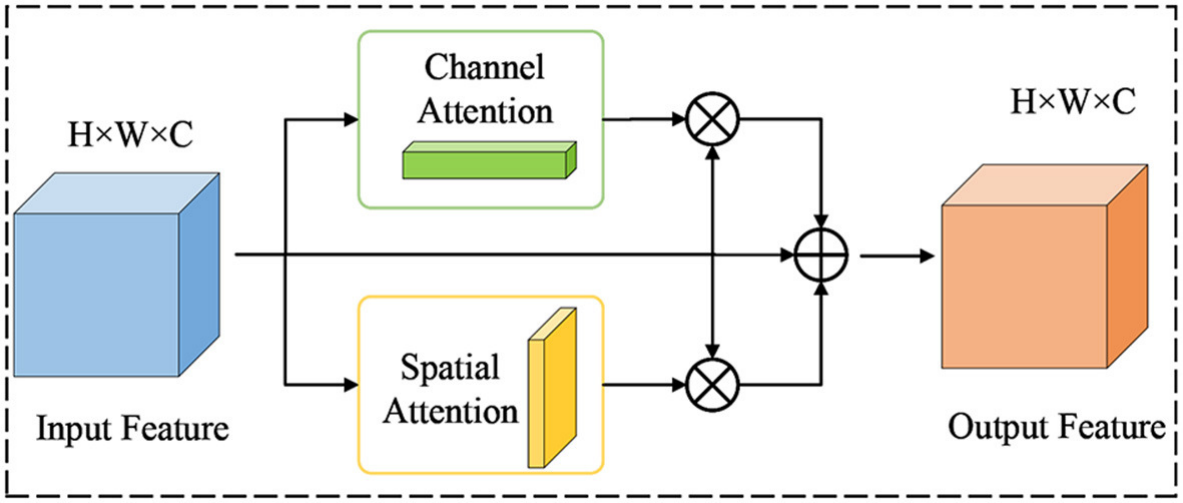
Illustration of the architecture of the P-CBAM attention module.

In neural networks, some layers are more concerned with channel features, while introducing spatial features can sensitize the network and even produce a lot of non-pixel information. Some layers are more concerned with spatial features and introducing channel features can cause overfitting to the network. However, the serial information exchange approach of the CBAM module, which prioritizes channels first, ignores this situation. Therefore, to address this situation, this paper proposes a new attention mechanism known as P-CBAM and introduce it to the bottom of the backbone network. The motivation is to overcome the problem of network sensitization due to spatial attention that prevents the model from effectively extracting polyp features, as well as the problem of network overfitting due to channel attention. In the P-CBAM attention mechanism, spatial and channel attention are given the same priority for different feature maps, and then they are weighted and fused as a way of exchanging information on the channel and spatial aspects of the feature maps. The structure of P-CBAM attention is shown in Figure 5.

For the input feature *F*∈*R*^*W*×*H*×*C*^, the channel attention and spatial attention are computed as shown in Equations 1, 2:


(1) $$\begin{array}{l} M_{c}\left(F\right)=\sigma \left(Conv\left(avgpool\left(F\right)\right)+Conv\left(maxpool\left(F\right)\right)\right) \end{array}$$

(2) $$\begin{array}{l} M_{S}\left(F\right)=\sigma \left(f^{7\times 7}\left(Concat\left(\left[avgpool\left(F\right),maxpool\left(F\right)\right]\right)\right)\right) \end{array}$$

where $M_{c}\left(F\right)\in R^{1\times 1\times C}$, represents the output of the channel attention module, $M_{S}\left(F\right)\in R^{W\times H\times 1}$ represents the output of the spatial attention module, and σ denotes the sigmoid activation function. Based on the above formula, the final output characteristics of the P-CBAM module are shown in Equation 3:


(3) $$\begin{array}{l} F_{out}=\frac{\alpha_{1}\cdot F_{c}+\alpha_{2}\cdot F_{s}+\alpha_{3}\cdot F}{\alpha_{1}+\alpha_{2}+\alpha_{3}} \end{array}$$

where *F*_*c*_ and *F*_*s*_ are the channel attention feature map and the spatial attention feature map, respectively, and *F* is the input feature map. α_1_ is the weight of channel attention, α_2_ is the weight of spatial attention, and α_3_ is the weight of residual links. P-CBAM obtains the attention information through the channel and on the two-dimensional space from a peer-to-peer perspective and can extract polyp features more effectively.

### 3.3 Loss function

The loss function of YOLOv5 consists of three main components: classification Loss *L*_*cls*_, confidence loss *L*_*obj*_, and bounding box regression loss *L*_*box*_ (Zhu et al., 2023). The purpose of the classification loss is to measure the accuracy of the model in classifying polyps. The confidence loss serves to measure the accuracy of the model's prediction of whether the bounding box contains polyps or not. It measures the model's ability to distinguish polyps from the background. The bounding box regression loss is used to measure the accuracy of the model's location of the polyp bounding box. The loss function is specifically described below:


(4) $$\begin{array}{l} Loss=L_{cls}+L_{box}+L_{obj} \end{array}$$

YOLOv5 uses CIoU as the regression loss function for the bounding box. It contains three parts: the IoU loss of the area of the overlapping region between the predicted frame and the real frame, the loss of the normalized distance between the centroids, and the loss of the aspect ratio of the width and the height. The CIoU bounding-box loss function ensures that the aspect ratio of the predicted frame to the real frame is closer during the training process, which accelerates the convergence of the bounding-box regression. However, IoU is more sensitive to the positional offset of the bounding box for small targets and is not suitable for small targets. In receiving rectal images, the size of polyps is generally small, and the offset of the prediction frame may cause non-overlap in the face of small-sized polyps. Therefore, this paper proposes to use the GWD (Gaussian Wasserstein Distance) loss as the regression loss of the bounding box.

The GWD loss is modeled as a two-dimensional Gaussian distribution for the prediction frame and the true frame, which are transformed into the prediction distribution $A\sim N\left(\mu_{a},\underset{a}{\sum }\right)$ and the truth distribution $B\sim N\left(\mu_{b},\underset{b}{\sum }\right)$, respectively, and the IoU between the prediction frame and the true frame is transformed into the similarity between the two distributions. Then the Wasserstein distance is used to compute the similarity between the two. In contrast to IoU, this method is not affected by polyp size.

For smaller-sized polyps, their pixels are mainly concentrated in the center of the bounding box, and the background pixels are distributed around the bounding box. The GWD loss is based on the bounding box to construct its inner tangent ellipse, and the specific details are shown in Equation 5:


(5) $$\begin{array}{l} \frac{{\left(x-c_{x}\right)}^{2}}{{\left(w/2\right)}^{2}}+\frac{{\left(y-c_{y}\right)}^{2}}{{\left(h/2\right)}^{2}}=1 \end{array}$$

where *c*_*x*_, *c*_*y*_ represent the center points of the bounding box and (*w, h*) represent the width and height of the bounding box. The 2D Gaussian distribution function is:


(6) $$\begin{array}{l} f\left(x|\mu ,\sum \right)=\frac{exp\left(-\frac{1}{2}{\left(x-\mu \right)}^{T}\sum \left(x-\mu \right)\right)}{2\pi |\sum {|}^{\frac{1}{2}}} \end{array}$$

where, when (*x*−μ)^*T*^∑(*x*−μ) = 1 is satisfied, *x* denotes the location information of the bounding box, μ denotes the mean and ∑ denotes the covariance matrix. After completing the 2D Gaussian modeling of the predicted and real bounding box use the Wasserstein distance to calculate the similarity between the two, the details of which are shown in Equation 7:


(7) $$W_{2}^{2}(A,B)={\left\|\mu_{a}-\mu_{b}\right\|}_{2}^{2}+{\left\|\sum_{a}^{1/2}-\sum_{b}^{1/2}\right\|}_{F}^{2}$$

## 4 Experimental result and analysis

### 4.1 Dataset

**Figure 6:**
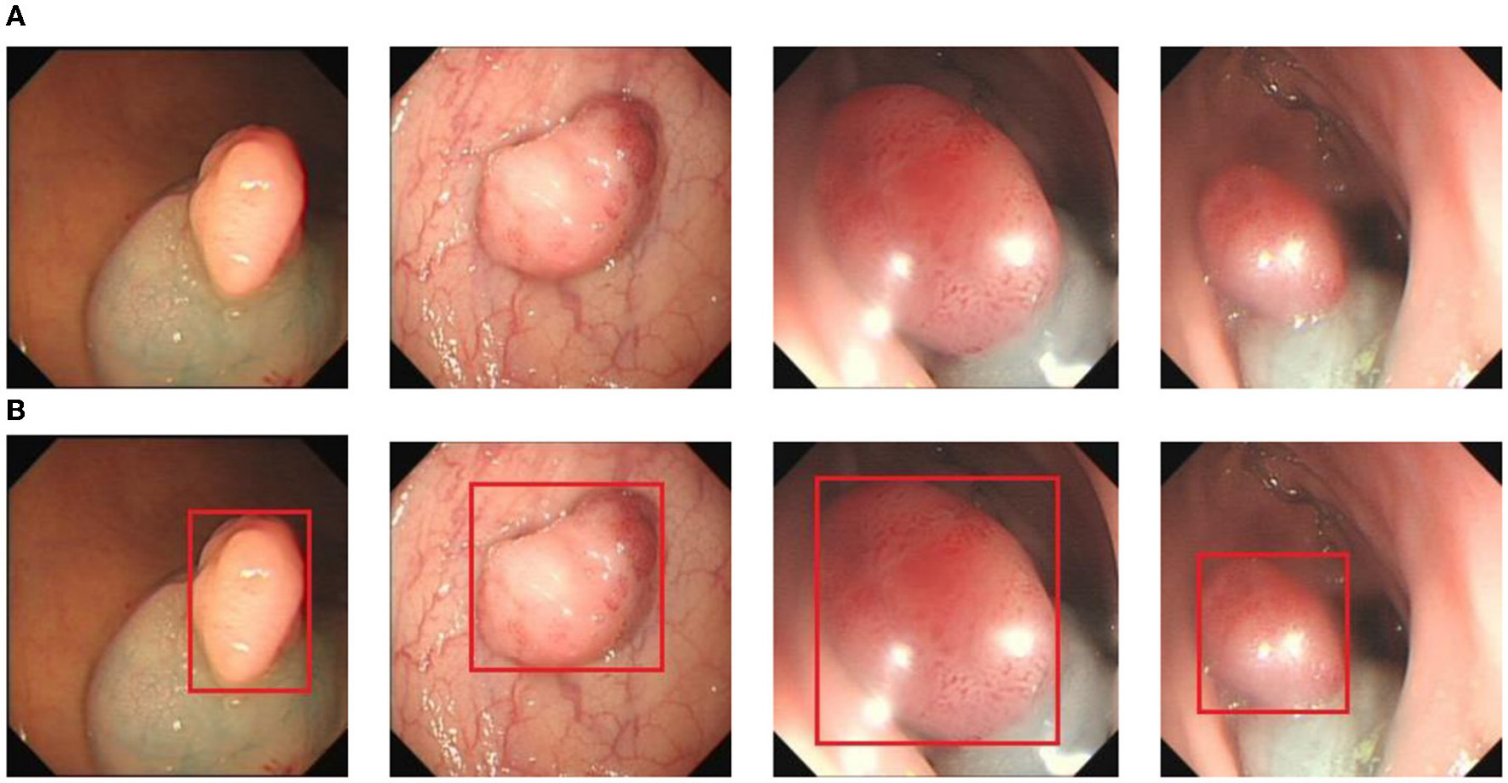
The polyp detection dataset. **(A)** Polyp images samples. **(B)** Ground truth with bounding box.

To obtain the data, we collected 1,200 images of colon cancer polyps at a local hospital and completed the labeling of the polyps using LabelImg. The labeled data format was converted to YOLO text format, and the categories, centroid coordinates, and width and height were generated and normalized. The dataset was divided into training, validation, and test sets in the ratio of 8:1:1. Figure 6 shows some example images of polyps, where Figure 6A is the polyp image data and Figure 6B is the ground truth box data of the labeled polyp.

### 4.2 Evaluation metrics

In this study, *Precision*, *Recall*, and *F*1−*score* were used as evaluation metrics for polyp object detection models. *Precision* measures the accuracy of the model in positive category prediction. It refers to how many of the samples that the model determines to be in the positive category are truly positive samples, which is described in detail as:


(8) $$\begin{array}{l} Precision=\frac{TP}{FP+TP} \end{array}$$

where *TP* denotes a true positive sample, i.e., the model correctly determines the target of a positive category as a positive category. *FP* denotes a false positive sample, i.e., the model incorrectly determines the target of a negative category as a positive category. *FN* denotes a false negative sample, i.e., the model incorrectly determines the target of a positive category as a negative category.

*Recall* measures the model's ability to detect positive category targets. It is how many of all positive category targets present in the dataset are successfully detected. A higher recall indicates that the model can detect more positive samples and reduce the number of polyps missed detection. It is described in detail as:


(9) $$\begin{array}{l} Recall=\frac{TP}{FN+TP} \end{array}$$

In the detection process, *Recall* and *Precision* usually have a trade-off relationship, where an increase in one side may lead to a decrease in the other side. Therefore, a trade-off between *Recall* and *Precision* is needed in the detection process. *F*1−*score* is a reconciled average of Precision and Recall, which is used to comprehensively evaluate the performance of the model. It helps to find the balance between Precision and Recall. Its detailed description is:


(10) $$\begin{array}{l} F1-score=\frac{2\times \left(Precision\times Recall\right)}{Precision+Recall} \end{array}$$

### 4.3 Ablation experiments and analysis

To further validate the detection performance of the methods proposed in this study and to understand the effect of different methods on the model, we conducted ablation experiments, the details of which are shown in Table 1.

**Table 1:** Impact of individual components in the development of model.

**Method**	**CBAM**	**P-CBAM**	**GWD**	**Precision**	**Recall**	**F1-Score**
YOLOv5				0.871	0.877	0.874
YOLOv5+CBAM	✓			0.878	0.886	0.882
YOLOv5+P-CBAM		✓		0.882	0.890	0.886
YOLOv5+GWD			✓	0.879	0.895	0.887
YOLOv5+P-CBAM+GWD		✓	✓	0.885	0.913	0.899

From Table 1, it can be seen that compared to the CBAM attention mechanism, our proposed P-CBAM attention mechanism can show better results. Compared to the initial model, the model with P-CBAM improves the precision value by 1.1% and the recall value by 1.3%. This proves that this attentional parallel processing approach addresses the shortcomings associated with the serial information exchange approach of initial attentional channel prioritization, and can effectively focus on deep polyp features. The introduction of GWD loss improves the model's precision value by 0.8% and recall value by 1.8%. This proves that the GWD improves the model's ability to regress on the target bounding box, allowing the model to exhibit higher accuracy. Introducing both the attention mechanism and GWD loss into the model, the precision was improved by 1.4% to 0.885, and the recall was improved by 3.6% to 0.913, which greatly improved the polyp detection rate. It proves the effectiveness of the method proposed in this paper in reducing the polyp missed detection rate.

### 4.4 Contrast experiments

To verify the effectiveness of the method proposed in this paper, we compare it with some mainstream single-stage and two-stage target detection algorithm models, and the experimental results are shown in Table 2.

**Table 2:** Performance of polyp detection between different algorithms.

**Method**	**Precision**	**Recall**	**F1-Score**
**R-CNN**	**0.907**	**0.889**	**0.897**
Faster R-CNN	**0.914**	0.896	0.905
YOLOv4	0.881	0.879	0.880
YOLOv7	0.783	0.764	0.773
YOLOv8	0.904	0.878	0.891
RT-DETR-R50	0.868	0.872	0.870
Ours	0.885	**0.913**	**0.899**

The bold values indicate that the optimal results obtained by each index.

From Table 2, our model exhibits the highest value in recall compared to other models, indicating that ours has the lowest miss-detection rate. Compared to the two-stage model Faster R-CNN, recall improves by 1.7%, and compared to the single-stage YOLO, recall improves by 3.4%. Combining detection accuracy and detection speed, our model shows better performance.

### 4.5 Visualization analysis of polyp detection

**Figure 7:**
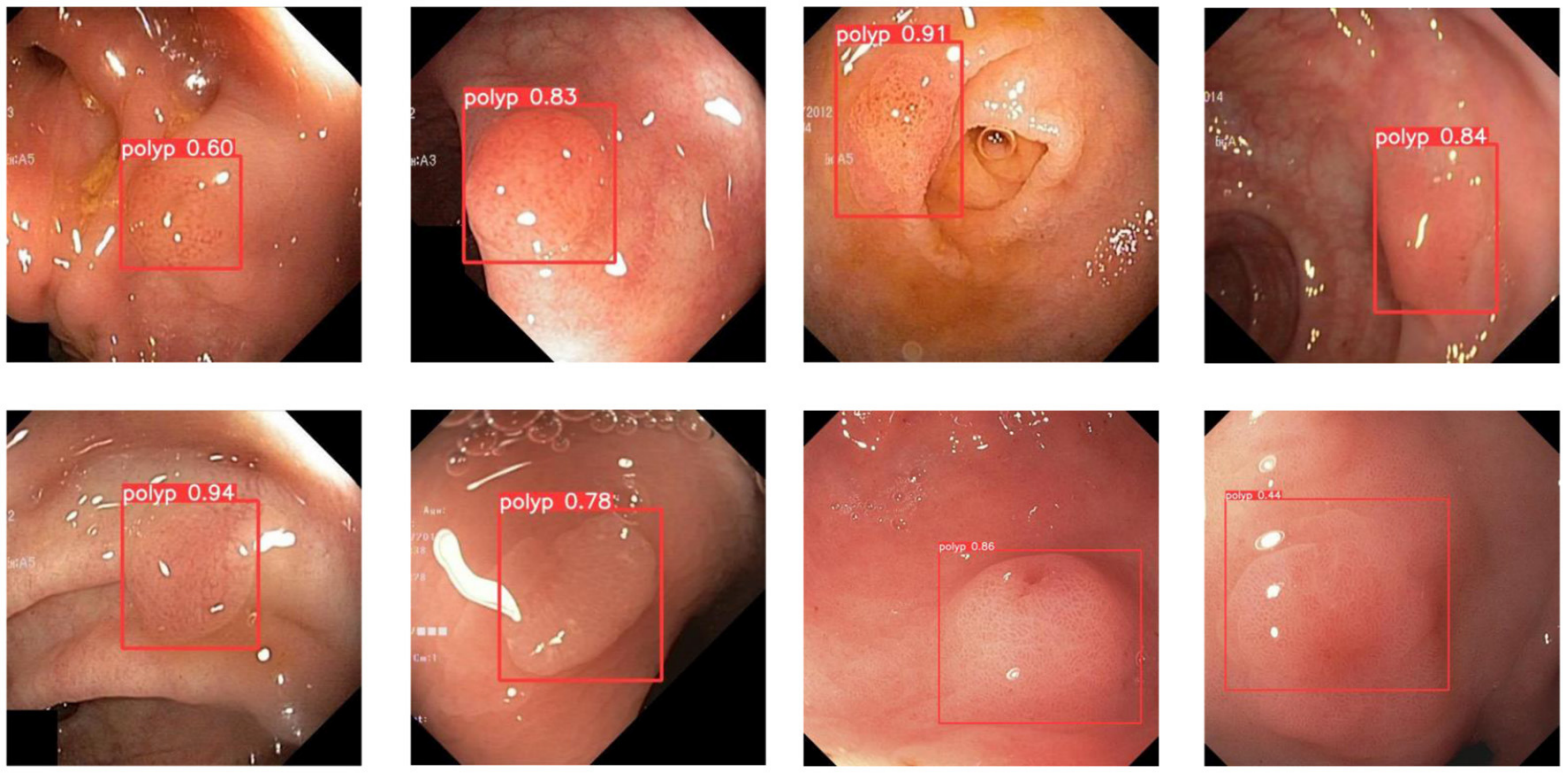
A subset of the detection results of polyps showing low contrasts to the background.

**Figure 8:**
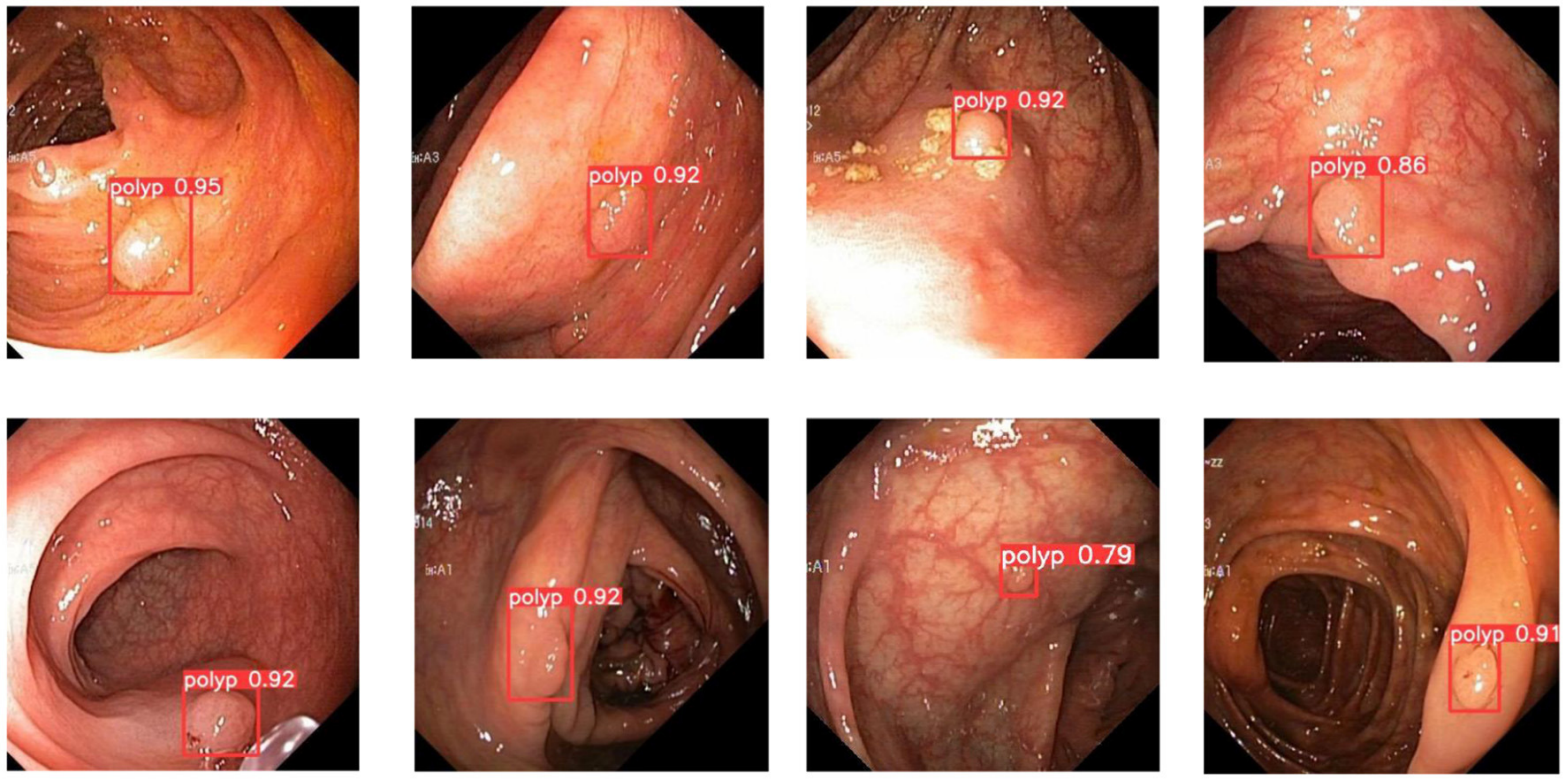
A subset of the detection results of small object polyps.

**Figure 9:**
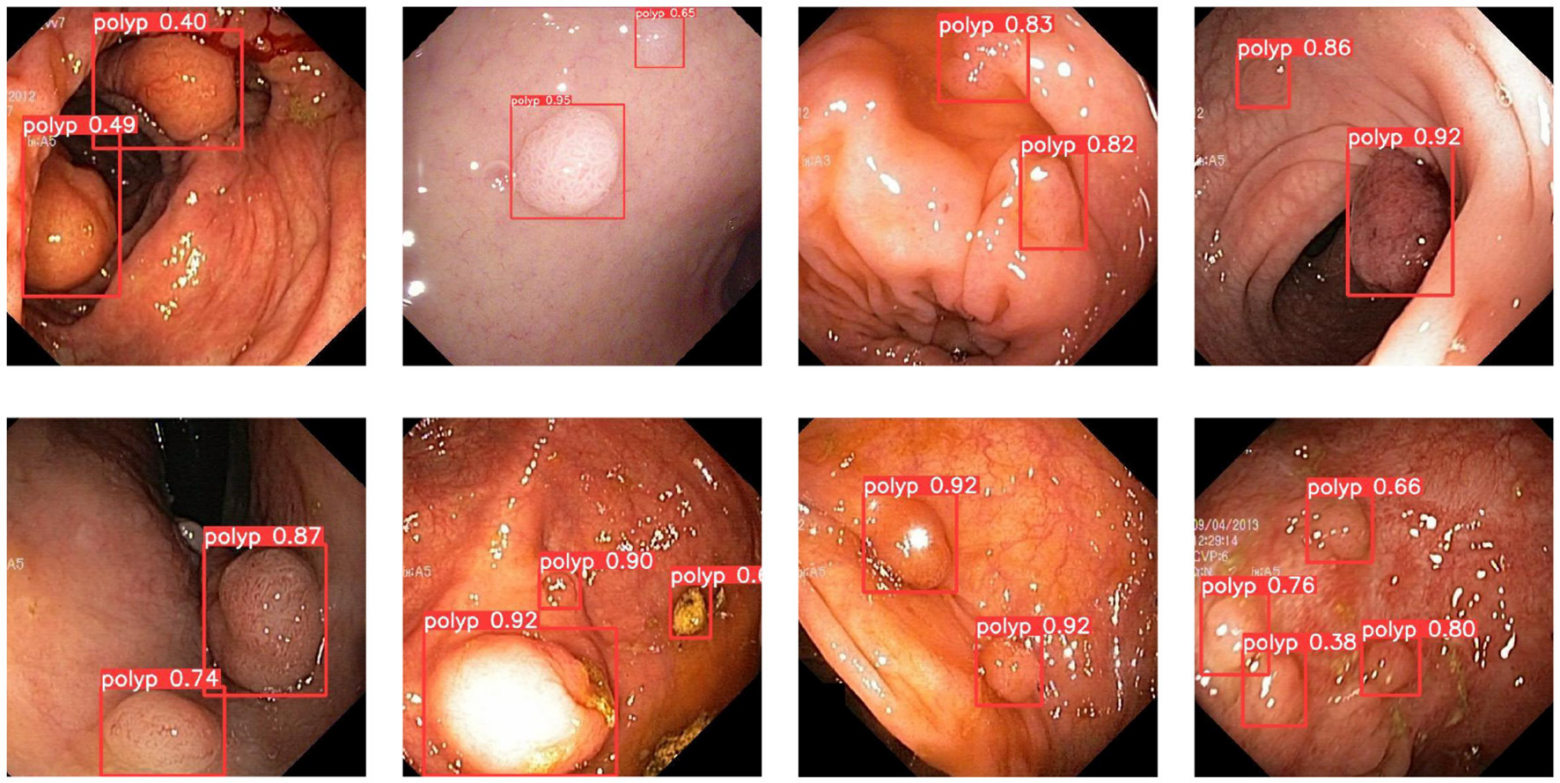
A subset of the detection results of multiple object polyps.

In order to demonstrate more intuitively the polyp detection effect of the improved model in different environments, we selected some representative polyp images in the dataset for visualization and comparison, the specific details of which are shown in Figures 7–9.

During colonoscopy, the color and texture of polyps are very similar to the surrounding circumstances in the intestine, which makes their detection more difficult. Figure 7 shows the detection of polyps with weak contrast with the background. As can be seen from the figure, the model proposed in this paper can detect polyps well even when facing polyps with weak contrast. This is because the attention mechanism can extract deeper semantic information about polyps, which makes the model pay more attention to polyp features.

In colorectal polyp detection, small polyps have been difficult to detect due to their tiny polyp characteristics. Figure 8 demonstrates what happens for the detection of small polyps. As can be seen from the figure, small polyps are accurately localized and identified. It can be proved that the use of GWD loss to calculate the similarity between the prediction bounding box and the ground truth box will not be affected by the size of the polyp and has strong robustness.

In addition, the improved model also shows good performance when faced with the presence of a larger number of polyps. Figure 9 demonstrates the detection of the model when dealing with multiple polyps. It can be seen that the model proposed in this paper can cope well with polyp detection in different environments.

## 5 Conclusions

Colorectal cancer is one of the most common malignant tumors in clinical practice, and the number of people suffering from it is on the rise every year. Effective diagnosis of colorectal cancer in the early stage can improve the recovery rate of patients. Colorectal cancer usually manifests as polyps in the early stage, therefore, the detection of polyps has become a necessary tool for the diagnosis of colorectal cancer. In the face of high leakage rates, this paper proposes an improved yolov5-based method that presents an attention mechanism to focus on polyp features and suppress non-critical information. In addition, a GWD loss is introduced to measure the similarity between the prediction box and grounding truth box. Experimental results demonstrate that the method exhibits precise localization and accurate recognition in the face of weak contrast of polyps to be detected, small polyps, and multiple polyps.

## Data availability statement

The raw data supporting the conclusions of this article will be made available by the authors, without undue reservation.

## Ethics statement

The studies involving humans were approved by Ethics Committee of Huai'an Second People's Hospital. The studies were conducted in accordance with the local legislation and institutional requirements. The participants provided their written informed consent to participate in this study. Written informed consent was obtained from the individual(s) for the publication of any potentially identifiable images or data included in this article.

## Author contributions

P-CZ: Conceptualization, Data curation, Methodology, Software, Writing – original draft. J-JW: Conceptualization, Investigation, Methodology, Writing – original draft, Writing – review & editing. WS: Conceptualization, Methodology, Software, Writing – original draft. X-CM: Conceptualization, Software, Writing – review & editing. B-LC: Conceptualization, Funding acquisition, Methodology, Validation, Writing – original draft.
